# Temporal Trends and Social Inequalities in Adverse Childhood Experiences Among Children in the United States, 2016-2023

**DOI:** 10.1177/00333549261488342

**Published:** 2026-10-28

**Authors:** Jalal Uddin, Muhammad Zakir Hossin, Saleh Al Morshed Sadid, Campbell Smith, Drishti Deepchand, Mohammad Hajizadeh

**Affiliations:** 1 School of Health Administration, Dalhousie University, Halifax, NS, Canada; 2 School of Population and Public Health, Human Early Learning Partnership, University of British Columbia, Vancouver, BC, Canada; 3 Department of Sociology, University of Dhaka, Dhaka, Bangladesh; 4 Faculty of Medicine, Halifax, NS, Canada

**Keywords:** adverse childhood experiences, social inequalities, trends, United States

## Abstract

**Objective:**

Temporal changes in specific adverse childhood experiences (ACEs) and related inequalities remain unclear. We examined trends and social inequalities in age-standardized ACE prevalence among US children from 2016 through 2023.

**Methods:**

Data were drawn from the National Survey of Children’s Health (n = 309 425; ages 0-17 y). Age-standardized prevalence was estimated for 9 ACEs and 2 summary measures (any ACE and ≥4 ACEs). Differences in ACE prevalence were examined across key social and demographic characteristics, including children’s sex, race and ethnicity, parental nativity, parental education, and family income.

**Results:**

From 2016 through 2023, the prevalence of financial hardship (from 25.3% to 13.8%), parental divorce (from 24.5% to 20.7%), parental imprisonment (from 7.9% to 5.6%), any ACE (from 46.0% to 37.4%), and ≥4 ACEs (from 6.1% to 4.8%) showed statistically significant declines (all *P* for trend < .001). In contrast, the prevalence of parental mental illness (from 7.8% to 8.3%) and race and ethnicity–related discrimination (ranging from 3.6% to 4.4%) increased modestly. Black children consistently had higher ACE prevalence than non-Hispanic White children, particularly for discrimination (ranging from 9.1% to 14.9%), financial hardship (from 17.5% to 36.2%), and parental imprisonment (from 9.4% to 15.8%) during 2016-2023. Racial and ethnic and nativity gaps in parental imprisonment and financial hardship narrowed over time, although substantial differences remained. Across all years, children from lower-income and lower-education families consistently experienced higher ACE prevalence than their more advantaged counterparts.

**Conclusions:**

Despite modest reductions in some ACEs, social differences in ACEs persisted over time. Findings highlight the continued concentration of ACEs among socioeconomically disadvantaged children, underscoring the need for structural and policy interventions targeting upstream social determinants.

## Introduction

Adverse childhood experiences (ACEs) are potentially traumatic events that are strongly associated with health and social outcomes.^1^ Typically, ACEs include forms of childhood maltreatment and household dysfunction.^2^ More recently, ACE frameworks have expanded to include broader community-level and structural adversities, including exposure to community violence, bullying, socioeconomic disadvantage, racial discrimination, and child welfare involvement.^3,4^

ACEs are unequally distributed across sociodemographic groups, with a disproportionate burden among historically marginalized communities of color and socioeconomically disadvantaged groups.^5,6^ ACEs have long-term consequences for morbidity and mortality,^1,7^ drive massive economic costs,^8^ and contribute to intergenerational and socioeconomic inequities in health and development.^5,9^

Monitoring temporal trends in ACE prevalence and its social patterning remains critical for informing prevention efforts. Many national ACE studies have relied on adults’ retrospective recall of childhood experiences or have focused primarily on cumulative ACE scores, which may obscure heterogeneity in how specific ACE types evolve over time.^4,6,10^ In contrast, the National Survey of Children’s Health (NSCH) collects contemporaneous parent or caregiver reports of children’s lifetime ACE exposure, providing an opportunity to monitor recent population-level patterns during childhood. Nevertheless, most existing trend analyses have used cumulative ACE measures and have not examined temporal changes across individual ACE domains. Furthermore, most analyses do not account for age-related differences in children’s exposure time,^10^ which may inflate prevalence estimates in samples with older age distributions. The absence of age standardization in prior studies limits comparability across survey years and sociodemographic groups when the underlying age composition of the child population changes over time. Moreover, socioeconomic inequities are often assessed at a single time point using pooled multi-year samples, limiting insight into dynamic changes during periods of social and economic instability. Few studies have examined how these socioeconomic differences in various types of ACEs have changed over time. Using a consistent descriptive analytic approach aligned with public health surveillance methods,^11,12^ we examined age-standardized temporal trends in 9 individual ACEs and reported commonly used summary measures (any ACE and ≥4 ACEs) to facilitate comparability with previous research. We further assessed whether social inequalities in ACE exposure by race and ethnicity, parental nativity, and socioeconomic position have changed over time.

## Methods

### Study Population

This study included noninstitutionalized US children aged 0 to 17 years who participated in the NSCH 2016-2023. The NSCH is a nationally representative annual household survey administered by the US Census Bureau using an address-based random sampling frame. One child was randomly selected from eligible households, and a parent or caregiver completed a self-administered online or paper-based survey.^13^ We used data from 2016 onward to account for a major redesign that standardized weighting and sampling procedures, thereby enabling comparability across later survey years.^14^ All datasets were downloaded from the Data Resource Center (https://www.childhealthdata.org/). In the 2016-2023 NSCH, data on 334 708 children were reported by their parents or caregivers. A complete-case analysis was conducted, excluding 25 283 children (7.5%) with missing data on any ACE variables or sociodemographic characteristics, yielding an analytical sample of 309 425 children. This study used secondary, publicly available data, with deidentified participants. Because of this, the study was exempt from Institutional Review Board approval at Dalhousie University.

### Variables

The outcome variable of interest was ACE. In the NSCH, parents/caregivers were asked whether the child had ever experienced any of the following: (1) difficulty covering basic needs due to family income; (2) parent or guardian divorced or separated; (3) parent or guardian died; (4) parent or guardian served time in jail; (5) saw or heard parents or adults slap, hit, kick, or punch one another in the home; (6) was a victim of violence or witnessed violence in their neighborhood; (7) lived with anyone who was mentally ill, suicidal, or severely depressed; (8) lived with anyone who had an alcohol or drug abuse problem; and (9) treated or judged unfairly due to race and ethnicity. Except for the financial hardship question, all ACE measures were assessed with dichotomous response options. The financial hardship item was originally measured on an ordinal scale and dichotomized for the current analysis, with responses of “very often” or “somewhat often” coded as “yes” and “rarely” or “never” coded as “no.”^12,15^ Given the conceptual heterogeneity of ACE domains, we analyzed the 9 ACE items as separate outcomes. In addition, we constructed 2 summary indicators: any ACE and ≥4 ACEs. The “any ACE” measure captures overall exposure to childhood adversity, whereas the ≥4 ACE threshold is widely used in ACE research and national surveillance because it identifies children exposed to multiple adversities who are at substantially greater risk of adverse health, developmental, and behavioral outcomes.^1,16^ Including these summary indicators facilitates comparison with prior NSCH studies and the broader ACE literature.^12^

Sociodemographic characteristics included children’s sex (male or female), race and ethnicity (Hispanic, non-Hispanic White, non-Hispanic Black, and Other/multiracial, non-Hispanic), parental nativity (US-born, at least 1 foreign-born parent, and US-born children living with caregivers other than parents, referred to as “other parental nativity”), educational attainment of the parent/caregiver (less than a high school degree, a high school degree, some college degree, and a college degree or higher), and family income expressed as the federal poverty level (FPL; 0%-99%, 100%-199%, 200%-399%, and ≥400%). These sociodemographic characteristics were selected a priori based on prior literature on social determinants of children’s health^10,12^ and their consistent availability across NSCH survey years.

### Statistical Analysis

We assessed temporal trends in age-standardized ACE prevalence from 2016-2023, overall and across sociodemographic groups. Age-standardized prevalence estimates were obtained by direct standardization using the pooled NSCH population as the standard. We estimated year-specific prevalence rates for each ACE outcome by key sociodemographic factors, including sex, race and ethnicity, parental nativity, parental education, and family income. Differences in temporal trends across groups were assessed using interaction terms in survey-weighted age-adjusted models. Linear trends within a specific group were assessed by modeling survey year as a continuous variable.

To quantify changes over time, we calculated absolute differences (2016 vs 2023) and relative differences (percentage change from 2016) in age-standardized prevalence. Statistical significance was defined as a 2-sided *P* < .05. Given the large sample size, interpretation emphasized the magnitude, consistency, and public health relevance of observed differences rather than statistical significance alone. Per NSCH analytic guidelines,^17^ all analyses incorporated survey weights and design variables to produce nationally representative estimates. All analyses were performed using Stata version 18 (StataCorp).

## Results

### Overall Temporal Trends in ACE Prevalence

Overall, most age-standardized ACEs showed statistically significant declines over time, particularly financial hardship, parental divorce, parental imprisonment, any ACE, and ≥4 ACEs (Table 1), whereas a few ACEs showed smaller or inconsistent changes. The prevalence of financial hardship decreased from 25.3% in 2016 to 13.8% in 2023, representing a relative reduction of approximately 46% (*P*-trend < .001). Similarly, parental imprisonment declined from 7.9% to 5.6%, representing a relative reduction of about 29% (*P*-trend < .001), and parental divorce declined from 24.5% to 20.7% (*P*-trend < .001). The prevalence of ≥4 ACEs declined from 6.1% to 4.8% (*P*-trend < .001), and any ACE decreased from 46.0% to 37.4% (*P*-trend < .001). In contrast, the prevalence of race and ethnicity–related discrimination and parental mental illness showed a modest upward trend across years. Race and ethnicity–related discrimination increased from 3.6% in 2016 to 4.4% in 2023, and parental mental illness increased from 7.8% in 2016 to 8.3% in 2023. Trends were similar for both sexes across all ACEs (all *P* for interaction > .05; Table 1).

**Table 1.** Trends in the Age-Standardized Prevalence of ACEs, Overall and by Sex, United States, 2016-2023.^a^

ACE	2016	2017	2018	2019	2020	2021	2022	2023	Absolute difference^b^	Relative difference^c^	*P* for trend within group^d^	*P* for trend between group^e^
	% (95% CI)	% (95% CI)	% (95% CI)	% (95% CI)	% (95% CI)	% (95% CI)	% (95% CI)	% (95% CI)				
Financial hardship												
Overall	25.3 (23.3-27.2)	22.8 (20.8-24.7)	15.1 (13.2-17.1)	15.1 (13.1-17.0)	12.5 (10.5-14.5)	11.8 (9.8-13.7)	14.0 (12.0-15.9)	13.8 (11.8-15.7)	−11.5	−45.6	NA	
Male	26.0 (22.1-29.9)	23.5 (19.6-27.5)	15.1 (11.1-19.0)	14.4 (10.5-18.3)	12.2 (8.3-16.1)	12.4 (8.5-16.4)	14.1 (10.2-18.0)	14.0 (10.1-18.0)	−11.9	−46.0	<.001	<.001
Female	24.6 (20.6-28.5)	21.9 (18.0-25.9)	15.2 (11.2-19.1)	15.7 (11.8-19.7)	12.8 (8.9-16.8)	11.0 (7.1-15.0)	13.8 (9.9-17.7)	13.5 (9.5-17.4)	−11.1	−45.2	<.001	.72
Parental divorce												
Overall	24.5 (22.5-26.4)	23.0 (21.0-24.9)	23.1 (21.2-25.1)	22.9 (21.0-24.9)	22.8 (20.9-24.8)	21.7 (19.7-23.6)	21.5 (19.5-23.4)	20.7 (18.7-22.6)	−3.8	−15.6	NA	
Male	24.5 (20.6-28.4)	23.3 (19.4-27.2)	22.9 (19.0-26.8)	21.9 (17.9-25.8)	22.9 (18.9-26.8)	21.9 (18.0-25.8)	21.5 (17.6-25.5)	20.7 (16.8-24.7)	−3.8	−15.4	<.001	<.001
Female	24.4 (20.5-28.4)	22.6 (18.7-26.5)	23.3 (19.4-27.2)	24.1 (20.1-28.0)	22.8 (18.9-26.7)	21.4 (17.5-25.4)	21.4 (17.5-25.3)	20.6 (16.7-24.5)	−3.8	−15.7	<.001	.83
Death of parent												
Overall	3.2 (1.2-5.2)	3.4 (1.5-5.4)	3.1 (1.1-5.1)	2.9 (1.0-4.9)	2.8 (0.8-4.7)	2.9 (0.9-4.8)	2.8 (0.8-4.8)	2.9 (0.9-4.9)	−0.3	−9.6	NA	
Male	3.5 (−0.4 to 7.4)	3.4 (−0.5 to 7.3)	3.0 (−0.9 to 6.9)	2.5 (−1.4 to 6.4)	2.8 (−1.1 to 6.7)	3.0 (−0.9 to 7.0)	2.9 (−1.0 to 6.9)	2.9 (−1.0 to 6.9)	−0.6	−16.3	.12	.01
Female	2.9 (−1.0 to 6.8)	3.5 (−0.4 to 7.4)	3.2 (−0.7 to 7.1)	3.4 (−0.5 to 7.3)	2.7 (−1.2 to 6.7)	2.7 (−1.2 to 6.6)	2.6 (−1.3 to 6.6)	2.9 (−1.1 to 6.8)	0.0	−0.9	.03	.84
Parental imprisonment												
Overall	7.9 (5.9-9.8)	7.0 (5.1-9.0)	7.3 (5.4-9.3)	7.2 (5.2-9.1)	6.5 (4.6-8.5)	6.2 (4.2-8.2)	6.0 (4.1-8.0)	5.6 (3.7-7.6)	−2.2	−28.6	NA	
Male	7.9 (4.0-11.8)	7.7 (3.8-11.6)	7.3 (3.4-11.2)	6.5 (2.6-10.4)	6.9 (3.0-10.8)	6.3 (2.4-10.2)	6.1 (2.2-10.0)	5.4 (1.5-9.3)	−2.5	−31.9	<.001	<.001
Female	7.8 (3.9-11.7)	6.3 (2.4-10.3)	7.4 (3.5-11.3)	7.9 (4.0-11.8)	6.2 (2.3-10.1)	6.1 (2.2-10.0)	5.9 (2.0-9.9)	5.9 (1.9-9.8)	−2.0	−25.0	<.001	.33
Domestic violence												
Overall	5.5 (3.6-7.5)	4.9 (2.9-6.8)	5.7 (3.7-7.6)	5.5 (3.6-7.5)	5.1 (3.1-7.0)	4.7 (2.7-6.7)	5.3 (3.3-7.2)	4.8 (2.9-6.8)	−0.7	−12.5	NA	
Male	5.7 (1.8-9.6)	5.3 (1.4-9.2)	5.3 (1.4-9.3)	5.1 (1.2-9.0)	5.2 (1.3-9.1)	4.7 (0.7-8.6)	5.2 (1.3-9.1)	4.8 (0.9-8.7)	−0.9	−15.3	.07	.05
Female	5.4 (1.5-9.3)	4.4 (0.5-8.3)	6.0 (2.1-9.9)	6.0 (2.0-9.9)	5.0 (1.0-8.9)	4.7 (0.8-8.7)	5.3 (1.4-9.3)	4.9 (0.9-8.8)	−0.5	−9.4	.36	.44
Neighborhood violence												
Overall	3.7 (1.8-5.7)	3.7 (1.8-5.7)	4.0 (2.0-5.9)	4.0 (2.0-6.0)	3.8 (1.9-5.8)	3.5 (1.5-5.4)	3.7 (1.7-5.6)	3.6 (1.6-5.5)	−0.2	−4.4	NA	
Male	3.7 (−0.2 to 7.6)	3.9 (−0.0 to 7.8)	3.9 (−0.1 to 7.8)	4.0 (0.1-8.0)	3.9 (−0.1 to 7.8)	3.2 (−0.7 to 7.1)	3.7 (−0.3 to 7.6)	3.6 (−0.3 to 7.5)	−0.1	−2.8	.28	.28
Female	3.7 (−0.2 to 7.6)	3.5 (−0.4 to 7.4)	4.1 (0.1-8.0)	3.9 (0.0-7.9)	3.8 (−0.1 to 7.7)	3.8 (−0.2 to 7.7)	3.7 (−0.2 to 7.6)	3.5 (−0.4 to 7.4)	−0.2	−5.9	.67	.64
Parental mental illness												
Overall	7.8 (5.8-9.7)	7.0 (5.0-9.0)	7.6 (5.6-9.5)	8.7 (6.8-10.7)	8.2 (6.2-10.1)	7.8 (5.8-9.8)	8.8 (6.8-10.8)	8.3 (6.3-10.3)	0.5	6.7	NA	
Male	7.5 (3.5-11.4)	7.2 (3.3-11.2)	7.2 (3.3-11.1)	8.7 (4.8-12.6)	8.1 (4.1-12.0)	7.4 (3.5-11.3)	8.5 (4.6-12.5)	8.3 (4.4-12.2)	0.9	11.5	.01	<.001
Female	8.1 (4.2-12.0)	6.7 (2.8-10.6)	8.0 (4.1-11.9)	8.8 (4.8-12.7)	8.3 (4.4-12.2)	8.2 (4.3-12.1)	9.1 (5.2-13.0)	8.3 (4.4-12.2)	0.2	2.2	.01	.96
Parental drug use												
Overall	9.0 (7.0-10.9)	7.8 (5.8-9.7)	7.9 (6.0-9.9)	8.7 (6.7-10.7)	8.4 (6.4-10.3)	7.8 (5.9-9.8)	8.2 (6.2-10.1)	7.8 (5.8-9.7)	−1.2	−13.5	NA	
Male	8.5 (4.6-12.4)	8.3 (4.4-12.2)	7.7 (3.8-11.7)	8.4 (4.5-12.4)	8.2 (4.3-12.1)	7.5 (3.5-11.4)	8.0 (4.0-11.9)	7.8 (3.9-11.7)	−0.7	−8.4	.14	.06
Female	9.4 (5.5-13.4)	7.2 (3.3-11.2)	8.1 (4.2-12.1)	8.9 (5.0-12.9)	8.6 (4.7-12.5)	8.2 (4.2-12.1)	8.4 (4.5-12.3)	7.7 (3.8-11.6)	−1.7	−18.4	.26	.82
Race and ethnicity–related discrimination												
Overall	3.6 (1.6-5.6)	3.7 (1.7-5.6)	3.9 (1.9-5.8)	4.7 (2.7-6.6)	5.3 (3.3-7.2)	4.6 (2.7-6.6)	4.2 (2.2-6.1)	4.4 (2.5-6.4)	0.8	22.8	NA	
Male	3.5 (−0.5 to 7.4)	3.9 (−0.0 to 7.8)	3.7 (−0.3 to 7.6)	5.0 (1.1-9.0)	5.2 (1.3-9.1)	4.3 (0.4-8.2)	4.1 (0.1-8.0)	4.4 (0.5-8.3)	1.0	27.7	.02	<.001
Female	3.8 (−0.2 to 7.7)	3.4 (−0.5 to 7.3)	4.1 (0.1-8.0)	4.3 (0.4-8.2)	5.3 (1.4-9.2)	4.9 (1.0-8.9)	4.3 (0.4-8.2)	4.4 (0.5-8.4)	0.7	18.2	.002	.56
Any ACE												
Overall	46.0 (44.1-48.0)	43.7 (41.7-45.6)	39.7 (37.7-41.7)	39.8 (37.9-41.8)	39.3 (37.3-41.2)	37.5 (35.6-39.5)	38.7 (36.8-40.7)	37.4 (35.5-39.4)	−8.6	−18.7	NA	
Male	46.5 (42.5-50.4)	44.6 (40.7-48.5)	39.4 (35.5-43.4)	38.5 (34.6-42.5)	39.0 (35.1-43.0)	37.8 (33.9-41.7)	39.0 (35.1-42.9)	37.7 (33.7-41.6)	−8.8	−19.0	<.001	<.001
Female	45.6 (41.7-49.5)	42.7 (38.8-46.6)	40.0 (36.1-43.9)	41.2 (37.3-45.1)	39.5 (35.6-43.4)	37.2 (33.3-41.2)	38.5 (34.6-42.4)	37.2 (33.2-41.1)	−8.4	−18.5	<.001	.75
≥4 ACEs												
Overall	6.1 (4.1-8.0)	5.3 (3.3-7.2)	5.6 (3.6-7.5)	5.4 (3.5-7.4)	5.0 (3.0-6.9)	4.6 (2.6-6.6)	4.9 (3.0-6.9)	4.8 (2.8-6.8)	−1.3	−21.0	NA	
Male	6.0 (2.1-10.0)	5.4 (1.5-9.4)	5.3 (1.4-9.2)	5.2 (1.3-9.1)	5.3 (1.3-9.2)	4.2 (0.3-8.1)	4.8 (0.8-8.7)	4.9 (1.0-8.8)	−1.2	−19.2	<.001	<.001
Female	6.1 (2.2-10.1)	5.1 (1.2-9.0)	5.8 (1.9-9.8)	5.7 (1.8-9.6)	4.7 (0.8-8.6)	5.0 (1.1-9.0)	5.1 (1.2-9.0)	4.7 (0.8-8.6)	−1.4	−23.0	.003	.77

Abbreviations: ACE, adverse childhood experience; NA, not applicable.

a All prevalences are survey-weighted. Data source: US Census Bureau.^13^

b Absolute difference represents the difference between the prevalence estimate in 2016 and 2023.

c Relative difference, expressed as the percentage change between 2016 and 2023, is calculated by dividing the absolute difference by the 2016 estimate and multiplying by 100.

d *P* for trend within each sex was estimated from survey-weighted logistic regression models that included survey year as a continuous variable (year centered at 2016) and adjusted for child age group, testing whether ACE prevalence changed linearly over time within each sex group.

e *P* for trend overall was obtained from survey-weighted logistic regression models including survey year as a continuous variable (year centered at 2016) and adjusted for child age group, testing whether ACE prevalence changed linearly over time in the overall population. *P* for trend between sex was obtained from interaction terms between survey year and sex in survey-weighted logistic regression models, testing whether temporal trends differed between males and females.

### ACE Prevalence by Race and Ethnicity

Black children consistently had the highest prevalence of several ACEs despite substantial declines in financial hardship and parental imprisonment (Table 2). Financial hardship among Black children declined from 36.2% in 2016 to 19.0% in 2023 but remained higher than among White (11.0% in 2023) and Hispanic (17.9% in 2023) children. Similarly, parental imprisonment among Black children declined from 15.8% to 9.4%, with larger absolute reductions than in other groups. Trends differed across groups for financial hardship (*P* = .003) but not for parental imprisonment.

**Table 2.** Trends in Age-Standardized Prevalence of ACEs by Race and Ethnicity, United States, 2016-2023.^a^

ACE	2016	2017	2018	2019	2020	2021	2022	2023	Absolute difference^b^	Relative difference^c^	*P* for trend within group^d^	*P* for trend between group^e^
	% (95% CI)	% (95% CI)	% (95% CI)	% (95% CI)	% (95% CI)	% (95% CI)	% (95% CI)	% (95% CI)				
Financial hardship												.003
Hispanic	28.9 (26.9-30.8)	24.9 (22.9-26.8)	17.1 (15.1-19.0)	18.7 (16.7-20.7)	16.3 (14.3-18.2)	14.7 (12.8-16.7)	17.0 (15.1-19.0)	17.9 (15.9-19.9)	−11.0	−38.0	<.001	
White	21.6 (19.6-23.5)	20.3 (18.4-22.3)	13.0 (11.0-14.9)	12.3 (10.3-14.2)	9.2 (7.2-11.2)	8.9 (7.0-10.9)	11.2 (9.2-13.2)	11.0 (9.0-12.9)	−10.6	−49.2	<.001	
Black	36.2 (34.2-38.2)	30.0 (28.0-31.9)	19.6 (17.7-21.6)	20.2 (18.3-22.2)	19.9 (18.0-21.9)	17.5 (15.5-19.4)	21.4 (19.4-23.3)	19.0 (17.0-20.9)	−17.2	−47.6	<.001	
Other^f^	23.3 (21.3-25.3)	20.2 (18.2-22.2)	16.1 (14.1-18.0)	13.7 (11.7-15.7)	10.7 (8.8-12.7)	11.6 (9.7-13.6)	11.6 (9.6-13.6)	10.9 (8.9-12.8)	−12.4	−53.3	<.001	
Parental divorce												.54
Hispanic	26.4 (24.5-28.4)	23.6 (21.6-25.5)	22.7 (20.7-24.7)	23.5 (21.6-25.5)	25.1 (23.1-27.0)	21.9 (20.0-23.9)	23.1 (21.2-25.1)	22.1 (20.1-24.0)	−4.3	−16.5	.04	
White	22.4 (20.4-24.3)	21.3 (19.3-23.2)	22.4 (20.5-24.4)	21.6 (19.7-23.6)	21.5 (19.5-23.4)	20.4 (18.5-22.4)	20.2 (18.2-22.2)	19.4 (17.4-21.4)	−3.0	−13.4	<.001	
Black	34.1 (32.2-36.1)	32.4 (30.4-34.3)	29.3 (27.4-31.3)	31.2 (29.3-33.2)	29.1 (27.2-31.1)	29.3 (27.3-31.2)	27.2 (25.3-29.2)	27.8 (25.8-29.8)	−6.3	−18.6	.002	
Other^f^	19.2 (17.2-21.1)	18.6 (16.6-20.5)	20.6 (18.6-22.5)	18.0 (16.1-20.0)	17.0 (15.1-19.0)	18.1 (16.1-20.0)	17.8 (15.9-19.8)	15.7 (13.8-17.7)	−3.4	−18.0	.01	
Death of parent												.78
Hispanic	2.9 (1.0-4.9)	3.2 (1.2-5.2)	3.3 (1.3-5.3)	2.6 (0.6-4.5)	2.7 (0.7-4.6)	2.7 (0.8-4.7)	2.7 (0.7-4.6)	2.8 (0.9-4.8)	−0.1	−3.9	.42	
White	2.6 (0.7-4.6)	2.6 (0.7-4.6)	2.6 (0.6-4.5)	2.5 (0.6-4.5)	2.2 (0.3-4.2)	2.4 (0.4-4.3)	2.1 (0.2-4.1)	2.5 (0.6-4.5)	−0.1	−3.0	.09	
Black	6.3 (4.3-8.2)	7.5 (5.6-9.5)	5.4 (3.4-7.3)	5.6 (3.7-7.6)	5.1 (3.2-7.1)	5.1 (3.1-7.0)	6.2 (4.3-8.2)	4.5 (2.6-6.5)	−1.7	−27.7	.05	
Other^f^	3.0 (1.1-5.0)	2.8 (0.9-4.8)	2.6 (0.6-4.6)	2.5 (0.5-4.5)	2.7 (0.7-4.6)	3.1 (1.1-5.0)	2.2 (0.3-4.2)	2.8 (0.8-4.7)	−0.2	−8.2	.53	
Parental imprisonment												.13
Hispanic	8.1 (6.1-10.0)	5.5 (3.6-7.5)	7.1 (5.2-9.1)	4.9 (2.9-6.9)	6.1 (4.1-8.1)	4.7 (2.8-6.7)	5.8 (3.9-7.8)	5.1 (3.2-7.1)	−2.9	−36.4	.003	
White	6.1 (4.1-8.1)	5.8 (3.9-7.8)	6.2 (4.3-8.2)	6.2 (4.3-8.2)	5.6 (3.6-7.6)	5.6 (3.6-7.5)	4.9 (2.9-6.8)	5.1 (3.2-7.1)	−1.0	−16.0	<.001	
Black	15.8 (13.8-17.8)	13.7 (11.8-15.7)	11.8 (9.9-13.8)	15.8 (13.8-17.7)	11.0 (9.0-12.9)	11.8 (9.8-13.7)	11.6 (9.7-13.6)	9.4 (7.5-11.4)	−6.4	−40.4	<.001	
Other^f^	7.1 (5.1-9.1)	8.5 (6.5-10.4)	7.7 (5.8-9.7)	6.7 (4.8-8.7)	7.0 (5.0-8.9)	6.1 (4.1-8.1)	5.7 (3.7-7.7)	5.0 (3.0-6.9)	−2.1	−30.1	.003	
Domestic violence												.96
Hispanic	5.4 (3.4-7.4)	4.2 (2.2-6.1)	6.0 (4.0-8.0)	4.8 (2.8-6.7)	5.4 (3.5-7.4)	4.4 (2.5-6.4)	5.6 (3.6-7.5)	4.6 (2.7-6.6)	−0.8	−14.0	.71	
White	4.9 (3.0-6.9)	4.6 (2.6-6.5)	4.7 (2.8-6.7)	5.0 (3.0-6.9)	4.4 (2.4-6.4)	4.2 (2.3-6.2)	4.5 (2.6-6.5)	4.3 (2.3-6.3)	−0.6	−12.4	.04	
Black	8.7 (6.7-10.7)	7.0 (5.1-9.0)	7.8 (5.8-9.8)	8.9 (6.9-10.9)	6.3 (4.3-8.3)	7.0 (5.0-9.0)	7.8 (5.8-9.7)	7.4 (5.4-9.4)	−1.3	−14.9	.45	
Other^f^	5.2 (3.2-7.1)	5.4 (3.4-7.3)	6.8 (4.9-8.8)	5.8 (3.8-7.8)	6.2 (4.2-8.2)	5.0 (3.0-6.9)	5.4 (3.4-7.3)	4.8 (2.8-6.8)	−0.4	−7.5	.24	
Neighborhood violence												.06
Hispanic	4.3 (2.4-6.3)	3.4 (1.5-5.4)	4.7 (2.8-6.7)	4.0 (2.1-6.0)	4.5 (2.6-6.5)	3.6 (1.7-5.6)	4.5 (2.6-6.5)	4.3 (2.3-6.3)	−0.1	−1.2	.79	
White	2.7 (0.7-4.6)	3.0 (1.0-5.0)	2.8 (0.8-4.7)	3.0 (1.1-5.0)	2.7 (0.7-4.6)	2.9 (1.0-4.9)	2.7 (0.8-4.7)	2.9 (0.9-4.9)	0.2	9.4	.89	
Black	6.5 (4.6-8.5)	6.6 (4.7-8.6)	6.7 (4.8-8.7)	8.2 (6.2-10.1)	7.2 (5.2-9.2)	5.5 (3.6-7.5)	6.2 (4.2-8.1)	5.0 (3.1-7.0)	−1.5	−22.9	.09	
Other^f^	4.4 (2.5-6.4)	4.1 (2.2-6.1)	4.5 (2.6-6.5)	3.5 (1.5-5.5)	3.7 (1.8-5.7)	3.5 (1.5-5.5)	3.4 (1.4-5.3)	3.0 (1.0-4.9)	−1.4	−32.6	.01	
Parental mental illness												.44
Hispanic	6.2 (4.2-8.2)	6.1 (4.2-8.1)	5.3 (3.3-7.2)	7.6 (5.7-9.6)	7.0 (5.0-8.9)	5.3 (3.3-7.2)	7.6 (5.7-9.6)	7.0 (5.1-9.0)	0.8	13.1	.15	
White	8.8 (6.8-10.7)	7.2 (5.2-9.1)	8.9 (7.0-10.9)	9.6 (7.6-11.5)	8.9 (7.0-10.9)	9.5 (7.6-11.5)	9.8 (7.8-11.8)	9.5 (7.6-11.5)	0.8	8.7	<.001	
Black	6.5 (4.5-8.4)	7.1 (5.2-9.1)	7.3 (5.3-9.3)	8.5 (6.6-10.5)	7.9 (6.0-9.9)	7.4 (5.5-9.4)	7.8 (5.8-9.7)	7.0 (5.0-9.0)	0.5	8.4	.58	
Other^f^	7.8 (5.8-9.7)	8.1 (6.1-10.1)	6.7 (4.7-8.7)	7.6 (5.7-9.6)	7.8 (5.8-9.7)	6.3 (4.4-8.3)	8.5 (6.5-10.4)	7.6 (5.7-9.6)	−0.1	−1.4	.91	
Parental drug use												.10
Hispanic	8.9 (7.0-10.9)	6.6 (4.7-8.6)	6.2 (4.3-8.2)	9.1 (7.2-11.1)	7.7 (5.7-9.7)	7.4 (5.4-9.3)	8.1 (6.2-10.1)	7.2 (5.3-9.2)	−1.7	−19.3	.83	
White	9.4 (7.4-11.4)	8.5 (6.6-10.5)	9.3 (7.3-11.3)	8.9 (7.0-10.9)	9.5 (7.5-11.4)	8.8 (6.8-10.7)	9.0 (7.0-10.9)	8.9 (6.9-10.8)	−0.5	−5.7	.53	
Black	7.7 (5.8-9.7)	6.9 (4.9-8.9)	6.0 (4.0-7.9)	7.9 (5.9-9.8)	6.6 (4.7-8.6)	6.3 (4.3-8.2)	6.7 (4.8-8.7)	6.3 (4.3-8.2)	−1.4	−18.8	.36	
Other^f^	8.0 (6.0-10.0)	8.1 (6.2-10.1)	8.0 (6.0-9.9)	7.5 (5.5-9.4)	6.9 (4.9-8.8)	6.3 (4.3-8.2)	6.5 (4.5-8.5)	6.1 (4.2-8.1)	−1.9	−23.2	.01	
Race and ethnicity–related discrimination												.39
Hispanic	4.4 (2.5-6.4)	4.2 (2.2-6.1)	4.6 (2.7-6.6)	6.6 (4.6-8.5)	6.2 (4.2-8.1)	5.1 (3.1-7.1)	4.3 (2.3-6.2)	5.3 (3.4-7.3)	0.9	19.8	.33	
White	0.9 (−1.0 to 2.9)	1.1 (−0.9 to 3.0)	1.3 (−0.7 to 3.2)	1.3 (−0.7 to 3.3)	1.6 (−0.3 to 3.6)	1.1 (−0.8 to 3.1)	0.9 (−1.0 to 2.9)	1.1 (−0.8 to 3.1)	0.2	19.7	.78	
Black	9.8 (7.9-11.8)	9.6 (7.7-11.6)	9.1 (7.2-11.1)	11.2 (9.3-13.2)	14.9 (13.0-16.9)	12.8 (10.9-14.8)	12.2 (10.3-14.2)	10.2 (8.2-12.2)	0.4	3.8	.02	
Other^f^	7.8 (5.9-9.8)	7.7 (5.8-9.7)	8.1 (6.2-10.1)	8.4 (6.4-10.3)	9.0 (7.0-10.9)	10.3 (8.3-12.3)	9.1 (7.1-11.1)	9.2 (7.2-11.2)	1.4	17.6	.02	
Any ACE												.82
Hispanic	51.4 (49.4-53.4)	47.0 (45.0-48.9)	40.7 (38.8-42.7)	42.1 (40.1-44.0)	43.7 (41.8-45.7)	39.6 (37.6-41.6)	42.2 (40.3-44.2)	41.1 (39.2-43.1)	−10.3	−20.0	<.001	
White	40.6 (38.7-42.6)	38.8 (36.9-40.8)	36.8 (34.8-38.7)	35.8 (33.9-37.8)	34.7 (32.7-36.7)	33.1 (31.2-35.1)	33.7 (31.7-35.7)	33.2 (31.2-35.1)	−7.4	−18.3	<.001	
Black	62.9 (60.9-64.8)	60.4 (58.5-62.4)	52.5 (50.6-54.5)	54.9 (52.9-56.9)	54.0 (52.0-56.0)	52.2 (50.2-54.2)	56.1 (54.1-58.0)	50.2 (48.3-52.2)	−12.6	−20.1	<.001	
Other^f^	41.5 (39.5-43.4)	39.1 (37.2-41.1)	37.2 (35.2-39.1)	36.0 (34.0-37.9)	33.8 (31.8-35.7)	36.3 (34.3-38.3)	35.1 (33.2-37.1)	33.6 (31.6-35.5)	−7.9	−19.1	<.001	
≥4 ACEs												.39
Hispanic	5.8 (3.8-7.7)	4.6 (2.7-6.6)	5.1 (3.2-7.1)	5.2 (3.2-7.1)	4.6 (2.7-6.6)	3.6 (1.7-5.6)	4.9 (2.9-6.8)	4.3 (2.3-6.3)	−1.5	−25.4	.05	
White	5.4 (3.4-7.3)	4.7 (2.8-6.7)	5.1 (3.1-7.0)	4.8 (2.8-6.8)	4.2 (2.3-6.2)	4.5 (2.6-6.5)	4.4 (2.5-6.4)	4.7 (2.7-6.6)	−0.7	−13.2	.01	
Black	9.2 (7.3-11.2)	8.1 (6.1-10.1)	6.8 (4.9-8.8)	8.8 (6.9-10.8)	8.1 (6.1-10.1)	7.1 (5.1-9.1)	7.0 (5.0-9.0)	6.7 (4.7-8.7)	−2.5	−27.2	.06	
Other^f^	6.5 (4.6-8.5)	5.8 (3.8-7.7)	7.4 (5.5-9.4)	5.0 (3.1-7.0)	5.8 (3.8-7.8)	4.4 (2.4-6.3)	5.1 (3.1-7.0)	4.5 (2.6-6.5)	−2.0	−30.9	.003	

Abbreviation: ACE, adverse childhood experience.

a All prevalences are survey-weighted. Data source: US Census Bureau.^13^

b Absolute difference represents the difference between the prevalence estimate in 2016 and 2023.

c Relative difference, expressed as the percentage change between 2016 and 2023, is calculated by dividing the absolute difference by the 2016 estimate and multiplying by 100.

d *P* for trend within group tests whether ACE prevalence changed linearly over time within each race and ethnicity group.

e *P* for trend between group tests whether linear time trends in ACE prevalence differed across race and ethnicity groups.

f Other races include Asian, American Indian or Alaska Native, Native Hawaiian or Other Pacific Islander, and children reporting multiple races.

Neighborhood violence, domestic violence, and parental drug use showed minimal or inconsistent changes across racial and ethnic groups. In contrast, race and ethnicity–related discrimination and parental mental illness showed modest increases or remained relatively stable. Discrimination among Black children increased from 9.8% in 2016 to 14.9% in 2020 before declining to 10.2% in 2023. Parental mental illness increased among White children but remained relatively stable among Black children. Overall, racial and ethnic differences in several ACEs persisted over time.

### ACE Prevalence by Parental Nativity

Children living with caregivers other than parents (herein “other parental nativity”) and those with US-born parents had a higher prevalence of ACE compared with those with at least 1 foreign-born parent (Table 3). Differences were observed for several ACEs, including financial hardship, parental divorce, parental mental illness, and parental imprisonment. Financial hardship declined across all parental-nativity groups (all *P*-trend < .001) but remained highest among children in the “other parental nativity” group. Trends differed across nativity groups for financial hardship (*P* for interaction = .01).

**Table 3.** Trends in Age-Standardized Prevalence of ACEs by Parental Nativity, United States, 2016-2023.^a^

ACE	2016	2017	2018	2019	2020	2021	2022	2023	Absolute difference^b^	Relative difference^c^	*P* for trend within nativity^d^	*P* for trend between nativity^e^
	% (95% CI)	% (95% CI)	% (95% CI)	% (95% CI)	% (95% CI)	% (95% CI)	% (95% CI)	% (95% CI)				
Financial hardship												.01
All parents US-born	25.7 (23.8-27.7)	23.4 (21.4-25.3)	15.5 (13.6-17.5)	15.6 (13.7-17.6)	12.2 (10.3-14.2)	11.0 (9.0-13.0)	14.2 (12.2-16.1)	13.6 (11.7-15.6)	−12.1	−47.0	<.001	
Any parent foreign-born	22.3 (20.3-24.3)	19.1 (17.1-21.0)	13.7 (11.8-15.7)	12.9 (11.0-14.9)	12.2 (10.2-14.1)	13.4 (11.5-15.4)	12.6 (10.7-14.6)	13.3 (11.3-15.3)	−9.0	−40.3	<.001	
Other parental nativity^f^	34.9 (33.0-36.9)	32.4 (30.4-34.3)	16.8 (14.8-18.7)	18.4 (16.4-20.3)	17.3 (15.4-19.3)	13.7 (11.7-15.7)	19.4 (17.4-21.4)	19.4 (17.5-21.4)	−15.5	−44.3	<.001	
Parental divorce												.33
All parents US-born	25.2 (23.3-27.2)	24.1 (22.1-26.1)	24.7 (22.8-26.7)	24.8 (22.9-26.8)	24.3 (22.4-26.3)	23.5 (21.6-25.5)	23.2 (21.3-25.2)	22.4 (20.5-24.4)	−2.8	−11.2	<.001	
Any parent foreign-born	15.7 (13.7-17.7)	14.6 (12.7-16.6)	14.8 (12.8-16.8)	12.6 (10.7-14.6)	14.0 (12.1-16.0)	13.1 (11.1-15.0)	13.6 (11.7-15.6)	13.4 (11.4-15.3)	−2.4	−15.0	.02	
Other parental nativity^f^	57.4 (55.5-59.4)	47.8 (45.8-49.7)	44.2 (42.2-46.1)	49.1 (47.1-51.0)	51.5 (49.5-53.4)	46.0 (44.1-48.0)	44.6 (42.7-46.6)	45.5 (43.5-47.4)	−12.0	−20.8	.02	
Death of parent												.05
All parents US-born	3.2 (1.2-5.1)	3.3 (1.3-5.2)	2.9 (0.9-4.8)	2.7 (0.7-4.6)	2.3 (0.4-4.3)	2.6 (0.6-4.5)	2.6 (0.7-4.6)	2.7 (0.7-4.6)	−0.5	−16.0	.005	
Any parent foreign-born	1.5 (−0.5 to 3.4)	1.7 (−0.3 to 3.7)	2.0 (0.0-4.0)	1.5 (−0.4 to 3.5)	1.5 (−0.4 to 3.5)	1.7 (−0.2 to 3.7)	1.5 (−0.5 to 3.5)	1.7 (−0.2 to 3.7)	0.3	17.3	.88	
Other parental nativity^f^	12.5 (10.5-14.4)	13.4 (11.4-15.4)	12.2 (10.2-14.1)	13.6 (11.6-15.5)	15.6 (13.7-17.6)	14.3 (12.3-16.2)	13.4 (11.5-15.4)	15.0 (13.0-16.9)	2.5	20.3	.24	
Parental imprisonment												.82
All parents US-born	7.9 (5.9-9.8)	7.1 (5.2-9.1)	7.4 (5.4-9.3)	7.7 (5.7-9.6)	6.6 (4.6-8.6)	6.2 (4.3-8.2)	6.4 (4.4-8.3)	5.9 (3.9-7.8)	−2.0	−25.5	<.001	
Any parent foreign-born	2.9 (0.9-4.8)	1.8 (−0.2 to 3.8)	2.3 (0.4-4.3)	1.6 (−0.4 to 3.5)	2.1 (0.1-4.1)	2.2 (0.2-4.1)	1.9 (−0.0 to 3.9)	2.0 (0.1-4.0)	−0.8	−28.7	.30	
Other parental nativity^f^	32.6 (30.6-34.6)	29.9 (28.0-31.9)	32.5 (30.6-34.5)	27.6 (25.6-29.5)	31.0 (29.1-33.0)	29.2 (27.2-31.1)	27.1 (25.2-29.1)	27.4 (25.5-29.4)	−5.2	−15.8	.08	
Domestic violence												.38
All parents US-born	5.2 (3.2-7.1)	4.8 (2.9-6.8)	5.6 (3.6-7.5)	5.5 (3.5-7.4)	4.9 (3.0-6.9)	4.5 (2.6-6.5)	5.4 (3.4-7.3)	5.0 (3.0-6.9)	−0.2	−3.9	.53	
Any parent foreign-born	3.3 (1.4-5.3)	2.5 (0.6-4.5)	3.4 (1.5-5.4)	2.9 (0.9-4.8)	2.8 (0.9-4.8)	2.5 (0.6-4.5)	2.9 (1.0-4.9)	2.3 (0.4-4.3)	−1.0	−29.1	.15	
Other parental nativity^f^	21.2 (19.3-23.2)	16.3 (14.3-18.3)	18.2 (16.3-20.2)	18.4 (16.5-20.4)	19.6 (17.7-21.6)	19.8 (17.8-21.8)	18.8 (16.8-20.7)	20.8 (18.8-22.8)	−0.4	−2.1	.57	
Neighborhood violence												.97
All parents US-born	3.6 (1.7-5.6)	3.6 (1.6-5.6)	3.8 (1.9-5.8)	4.2 (2.3-6.2)	3.7 (1.8-5.7)	3.4 (1.4-5.4)	3.8 (1.8-5.7)	3.6 (1.6-5.5)	−0.1	−1.5	.70	
Any parent foreign-born	2.6 (0.6-4.5)	2.6 (0.7-4.6)	3.0 (1.0-5.0)	2.0 (−0.0 to 3.9)	2.8 (0.9-4.8)	2.6 (0.6-4.6)	2.4 (0.4-4.3)	2.5 (0.6-4.5)	0.0	−1.8	.74	
Other parental nativity^f^	10.9 (9.0-12.9)	10.4 (8.4-12.4)	10.4 (8.5-12.4)	10.9 (8.9-12.8)	11.1 (9.2-13.1)	10.1 (8.1-12.0)	11.0 (9.0-13.0)	10.9 (8.9-12.8)	0.0	−0.4	.97	
Parental mental illness												.40
All parents US-born	9.0 (7.1-11.0)	7.2 (5.2-9.1)	8.9 (6.9-10.9)	10.2 (8.2-12.1)	9.2 (7.2-11.1)	9.2 (7.3-11.2)	10.3 (8.3-12.3)	9.7 (7.8-11.7)	0.7	7.7	<.001	
Any parent foreign-born	2.9 (1.0-4.9)	5.1 (3.1-7.0)	3.0 (1.0-4.9)	3.5 (1.5-5.4)	4.3 (2.3-6.3)	3.1 (1.1-5.1)	3.9 (2.0-5.9)	3.6 (1.7-5.6)	0.7	24.3	.97	
Other parental nativity^f^	15.6 (13.7-17.6)	14.1 (12.2-16.1)	13.9 (11.9-15.8)	15.9 (14.0-17.9)	16.0 (14.1-18.0)	14.9 (12.9-16.8)	16.0 (14.1-18.0)	17.9 (16.0-19.9)	2.3	14.8	.20	
Parental drug use												.89
All parents US-born	9.2 (7.2-11.1)	8.4 (6.4-10.3)	8.5 (6.6-10.5)	9.2 (7.2-11.1)	9.0 (7.1-11.0)	8.3 (6.3-10.2)	8.9 (7.0-10.9)	8.5 (6.6-10.5)	−0.6	−7.1	.52	
Any parent foreign-born	4.4 (2.4-6.3)	2.8 (0.8-4.7)	3.4 (1.4-5.3)	4.0 (2.1-6.0)	3.4 (1.4-5.3)	3.4 (1.4-5.3)	3.5 (1.6-5.5)	3.4 (1.5-5.4)	−0.9	−21.6	.64	
Other parental nativity^f^	29.2 (27.2-31.1)	24.0 (22.1-26.0)	23.4 (21.5-25.4)	24.8 (22.9-26.8)	27.2 (25.2-29.1)	27.5 (25.6-29.5)	26.7 (24.7-28.6)	26.8 (24.9-28.8)	−2.3	−8.1	.74	
Race and ethnicity–related discrimination												.91
All parents US-born	3.3 (1.4-5.3)	3.7 (1.7-5.6)	3.5 (1.6-5.5)	4.5 (2.6-6.5)	4.8 (2.8-6.7)	4.3 (2.3-6.2)	4.2 (2.3-6.2)	4.0 (2.1-6.0)	0.7	21.2	<.001	
Any parent foreign-born	3.9 (1.9-5.9)	3.5 (1.5-5.5)	4.2 (2.2-6.1)	5.1 (3.1-7.0)	6.1 (4.1-8.1)	5.3 (3.4-7.3)	3.9 (2.0-5.9)	5.1 (3.1-7.0)	1.2	29.8	.03	
Other parental nativity^f^	5.8 (3.9-7.8)	4.2 (2.2-6.1)	6.3 (4.3-8.3)	4.5 (2.5-6.4)	7.1 (5.2-9.1)	5.4 (3.4-7.3)	5.2 (3.3-7.2)	6.4 (4.5-8.4)	0.6	10.2	.49	
Any ACE												.89
All parents US-born	46.1 (44.2-48.1)	44.8 (42.8-46.8)	41.2 (39.2-43.1)	41.5 (39.5-43.4)	40.2 (38.3-42.2)	38.2 (36.2-40.2)	40.2 (38.2-42.1)	38.8 (36.9-40.8)	−7.3	−15.9	<.001	
Any parent foreign-born	39.0 (37.0-40.9)	34.7 (32.8-36.7)	30.9 (28.9-32.8)	30.0 (28.0-32.0)	31.1 (29.2-33.1)	31.1 (29.1-33.0)	30.3 (28.3-32.2)	30.5 (28.5-32.4)	−8.5	−21.8	.01	
Other parental nativity^f^	79.0 (77.0-80.9)	71.6 (69.6-73.5)	65.2 (63.3-67.2)	66.4 (64.4-68.3)	71.2 (69.2-73.1)	66.1 (64.1-68.0)	71.2 (69.2-73.1)	65.0 (63.0-66.9)	−14.0	−17.7	.04	
≥4 ACEs												.57
All parents US-born	6.2 (4.2-8.2)	5.2 (3.2-7.2)	5.8 (3.9-7.8)	6.0 (4.0-8.0)	4.8 (2.9-6.8)	4.8 (2.9-6.8)	5.4 (3.4-7.4)	5.1 (3.1-7.1)	−1.1	−17.6	.004	
Any parent foreign-born	2.1 (0.2-4.1)	2.0 (0.0-4.0)	2.2 (0.2-4.1)	1.3 (−0.6 to 3.3)	2.3 (0.4-4.3)	1.4 (−0.5 to 3.4)	1.5 (−0.5 to 3.4)	1.6 (−0.4 to 3.6)	−0.5	−24.4	.08	
Other parental nativity^f^	24.7 (22.8-26.7)	21.0 (19.1-23.0)	19.3 (17.3-21.3)	18.0 (16.0-19.9)	22.3 (20.4-24.3)	20.3 (18.3-22.2)	19.8 (17.8-21.7)	23.2 (21.2-25.1)	−1.6	−6.4	.60	

Abbreviation: ACE, adverse childhood experience.

a All prevalences are survey-weighted. Data source: US Census Bureau.^13^

b The absolute difference represents the difference between the prevalence estimate in 2016 and 2023.

c Relative difference, expressed as the percentage change between 2016 and 2023, is calculated by dividing the absolute difference by the 2016 estimate and multiplying by 100.

d *P* for trend within group tests whether ACE prevalence changed linearly over time within each parental nativity group.

e *P* for trend between group tests whether linear time trends in ACE prevalence differed across parental nativity groups.

f Other parental nativity refers to children born in the United States who live with caregiver(s) other than parents.

Parental divorce declined across all groups, with larger reductions among children living with caregivers other than parents (−12 percentage points) compared with those with US-born parents (−2.8 percentage points) and those with at least 1 foreign-born parent (−2.4 percentage points). Despite these declines, parental divorce remained substantially higher among children living with caregivers other than parents (45.5%) compared with children with US-born parents (22.4%) and those with a foreign-born parent (13.4%) in 2023.

Similarly, declines in parental imprisonment were larger among children living with caregiver(s) other than parents (−5.2 percentage points) than among children of US-born parents (−2.0 percentage points) or of any foreign-born parents (−0.8 percentage points). In contrast, race and ethnicity–related discrimination and parental mental illness showed modest increases or remained stable, with no differences across parental-nativity groups (*P* for interaction ≥.05). The death of a parent changed modestly over time, with evidence that trends differed by parental nativity (*P* < .05).

Overall, trends differed across parental-nativity groups for financial hardship (*P* = .01) and parent death (*P* < .05) but not for other ACEs (*P* for interactions ≥.05).

### ACE Prevalence by Parental Education

Across all survey years, children with lower parental education levels consistently experienced a higher prevalence of most ACEs than those with higher parental education levels (Table 4).

**Table 4.** Trends in Age-Standardized Prevalence of ACEs by Parental Education, United States, 2016-2023.^a^

ACE	2016	2017	2018	2019	2020	2021	2022	2023	Absolute difference^a^	Relative difference^c^	*P* for trend within education^d^	*P* for trend between education^e^
	% (95% CI)	% (95% CI)	% (95% CI)	% (95% CI)	% (95% CI)	% (95% CI)	% (95% CI)	% (95% CI)				
Financial hardship												<.001
Less than high school	32.5 (30.5-34.5)	32.5 (30.5-34.4)	23.3 (21.3-25.3)	17.7 (15.8-19.7)	22.0 (20.0-23.9)	21.7 (19.7-23.7)	21.5 (19.5-23.5)	22.3 (20.3-24.2)	−10.2	−31.5	<.001	
High school or GED	37.3 (35.4-39.3)	31.3 (29.4-33.3)	22.9 (20.9-24.8)	24.9 (23.0-26.9)	21.0 (19.1-23.0)	19.8 (17.8-21.8)	22.6 (20.6-24.5)	24.4 (22.4-26.3)	−13.0	−34.7	<.001	
Some college or technical school	35.4 (33.4-37.4)	33.6 (31.6-35.6)	21.3 (19.4-23.3)	21.1 (19.2-23.1)	16.5 (14.5-18.5)	16.0 (14.1-18.0)	22.7 (20.7-24.6)	20.5 (18.5-22.5)	−14.9	−42.1	<.001	
College degree or higher	14.7 (12.8-16.7)	13.1 (11.1-15.1)	8.3 (6.4-10.3)	8.2 (6.3-10.2)	6.1 (4.1-8.0)	5.5 (3.5-7.5)	6.9 (4.9-8.8)	6.9 (4.9-8.8)	−7.8	−53.3	<.001	
Parental divorce												.92
Less than high school	27.1 (25.2-29.1)	20.5 (18.5-22.5)	21.6 (19.6-23.6)	22.3 (20.3-24.3)	23.5 (21.5-25.4)	19.1 (17.2-21.1)	25.0 (23.1-27.0)	21.4 (19.5-23.4)	−5.7	−21.1	.43	
High school or GED	34.4 (32.5-36.4)	33.2 (31.2-35.2)	31.6 (29.6-33.5)	32.6 (30.7-34.6)	32.2 (30.3-34.2)	30.3 (28.3-32.2)	30.7 (28.8-32.7)	31.0 (29.0-32.9)	−3.5	−10.1	.03	
Some college or technical school	33.3 (31.3-35.2)	30.3 (28.4-32.3)	31.4 (29.5-33.4)	31.5 (29.6-33.5)	31.1 (29.1-33.0)	31.8 (29.8-33.7)	28.9 (27.0-30.9)	27.9 (26.0-29.9)	−5.3	−16.0	.002	
College degree or higher	16.2 (14.2-18.1)	16.3 (14.3-18.2)	16.5 (14.6-18.5)	15.9 (13.9-17.8)	16.1 (14.2-18.1)	15.1 (13.2-17.1)	15.0 (13.1-17.0)	14.7 (12.7-16.6)	−1.5	−9.3	<.001	
Death of parent												.94
Less than high school	4.4 (2.5-6.4)	3.0 (1.0-4.9)	3.0 (1.1-5.0)	4.0 (2.0-5.9)	3.4 (1.5-5.4)	1.8 (−0.2 to 3.7)	2.8 (0.9-4.8)	4.5 (2.5-6.4)	0.0	0.8	.41	
High school or GED	4.8 (2.9-6.8)	6.8 (4.8-8.8)	5.4 (3.4-7.4)	4.3 (2.4-6.3)	4.2 (2.2-6.1)	5.2 (3.2-7.1)	4.6 (2.7-6.6)	5.2 (3.3-7.2)	0.4	8.8	.33	
Some college or technical school	4.2 (2.3-6.2)	3.3 (1.3-5.2)	4.3 (2.3-6.2)	4.3 (2.3-6.3)	3.4 (1.4-5.3)	3.4 (1.4-5.3)	3.7 (1.7-5.7)	3.9 (1.9-5.8)	−0.4	−8.6	.47	
College degree or higher	1.8 (−0.2 to 3.7)	2.2 (0.3-4.2)	1.8 (−0.2 to 3.7)	1.6 (−0.4 to 3.6)	1.8 (−0.1 to 3.8)	2.0 (0.1-4.0)	1.8 (−0.2 to 3.7)	1.5 (−0.4 to 3.5)	−0.2	−13.7	.14	
Parental imprisonment												.75
Less than high school	9.6 (7.6-11.5)	9.3 (7.3-11.2)	11.0 (9.1-13.0)	8.1 (6.1-10.0)	7.3 (5.4-9.3)	7.8 (5.8-9.7)	8.0 (6.1-10.0)	6.7 (4.7-8.6)	−2.9	−30.2	.07	
High school or GED	13.7 (11.7-15.6)	13.6 (11.7-15.6)	13.3 (11.3-15.2)	13.5 (11.5-15.4)	13.0 (11.0-15.0)	10.9 (9.0-12.9)	10.6 (8.6-12.6)	10.9 (8.9-12.8)	−2.8	−20.5	<.001	
Some college or technical school	12.4 (10.4-14.3)	9.1 (7.1-11.0)	10.4 (8.4-12.4)	11.3 (9.3-13.2)	9.9 (7.9-11.8)	9.8 (7.8-11.7)	9.4 (7.5-11.4)	8.5 (6.5-10.4)	−3.9	−31.5	<.001	
College degree or higher	3.1 (1.2-5.1)	3.1 (1.2-5.1)	3.1 (1.2-5.1)	3.0 (1.0-5.0)	2.7 (0.7-4.6)	2.7 (0.8-4.7)	2.8 (0.8-4.8)	2.7 (0.7-4.6)	−0.5	−15.2	.03	
Domestic violence												.90
Less than high school	5.7 (3.8-7.7)	4.7 (2.7-6.6)	6.7 (4.8-8.7)	4.8 (2.8-6.8)	5.1 (3.2-7.1)	4.8 (2.9-6.8)	7.2 (5.3-9.2)	3.9 (2.0-5.9)	−1.8	−31.8	.70	
High school or GED	8.5 (6.6-10.5)	6.7 (4.8-8.7)	7.6 (5.6-9.6)	7.6 (5.6-9.5)	7.0 (5.0-9.0)	6.0 (4.1-8.0)	8.0 (6.1-10.0)	7.5 (5.5-9.4)	−1.0	−12.1	.56	
Some college or technical school	7.7 (5.8-9.7)	7.1 (5.1-9.0)	8.5 (6.5-10.4)	9.6 (7.6-11.6)	7.9 (6.0-9.9)	7.2 (5.2-9.1)	7.5 (5.5-9.4)	8.1 (6.2-10.1)	0.4	5.1	.99	
College degree or higher	3.3 (1.3-5.2)	3.2 (1.2-5.2)	3.5 (1.6-5.5)	3.2 (1.2-5.1)	3.2 (1.2-5.1)	3.2 (1.2-5.1)	3.2 (1.2-5.1)	2.9 (1.0-4.9)	−0.4	−10.7	.25	
Neighborhood violence												.22
Less than high school	5.9 (4.0-7.9)	3.3 (1.4-5.3)	3.9 (2.0-5.9)	4.4 (2.4-6.3)	6.1 (4.2-8.1)	6.5 (4.6-8.5)	5.1 (3.2-7.1)	6.5 (4.6-8.5)	0.6	10.2	.21	
High school or GED	4.7 (2.7-6.7)	4.7 (2.8-6.7)	6.5 (4.5-8.4)	6.5 (4.5-8.4)	5.5 (3.5-7.5)	4.1 (2.1-6.0)	6.2 (4.2-8.1)	4.7 (2.8-6.7)	0.0	0.6	.98	
Some college or technical school	5.5 (3.5-7.4)	5.6 (3.6-7.5)	6.2 (4.2-8.1)	5.5 (3.6-7.5)	5.6 (3.6-7.5)	4.5 (2.6-6.5)	4.9 (2.9-6.8)	5.0 (3.1-7.0)	−0.4	−8.0	.07	
College degree or higher	2.0 (0.1-4.0)	2.4 (0.5-4.4)	2.1 (0.1-4.0)	2.3 (0.3-4.2)	2.1 (0.1-4.0)	2.3 (0.3-4.2)	2.1 (0.2-4.1)	2.2 (0.2-4.1)	0.1	6.8	.87	
Parental mental illness												.42
Less than high school	5.6 (3.6-7.5)	2.7 (0.7-4.7)	5.3 (3.3-7.3)	6.1 (4.2-8.1)	6.5 (4.5-8.4)	3.7 (1.7-5.7)	6.1 (4.1-8.1)	5.2 (3.3-7.2)	−0.4	−6.6	.31	
High school or GED	8.8 (6.9-10.8)	7.5 (5.6-9.5)	9.4 (7.5-11.4)	10.2 (8.2-12.1)	8.8 (6.9-10.8)	6.9 (4.9-8.8)	9.9 (7.9-11.9)	9.4 (7.5-11.4)	0.6	6.5	.49	
Some college or technical school	9.6 (7.6-11.6)	8.7 (6.8-10.7)	9.4 (7.4-11.4)	11.6 (9.6-13.5)	10.5 (8.6-12.5)	11.2 (9.2-13.1)	12.0 (10.0-13.9)	10.9 (8.9-12.8)	1.3	13.3	<.001	
College degree or higher	7.0 (5.0-8.9)	6.7 (4.8-8.7)	6.6 (4.6-8.6)	7.4 (5.5-9.4)	7.4 (5.4-9.3)	7.6 (5.7-9.6)	7.7 (5.7-9.6)	7.5 (5.6-9.5)	0.6	8.3	.008	
Parental drug use												.80
Less than high school	7.9 (6.0-9.9)	6.5 (4.5-8.4)	5.6 (3.7-7.6)	7.0 (5.0-8.9)	6.5 (4.5-8.4)	5.6 (3.6-7.6)	5.9 (3.9-7.8)	8.2 (6.2-10.2)	0.3	3.4	.75	
High school or GED	12.1 (10.1-14.0)	11.3 (9.3-13.3)	11.2 (9.2-13.2)	12.8 (10.8-14.7)	10.5 (8.5-12.4)	9.9 (7.9-11.8)	11.8 (9.8-13.7)	11.0 (9.0-12.9)	−1.1	−9.1	.35	
Some college or technical school	12.3 (10.3-14.2)	9.6 (7.7-11.6)	11.6 (9.7-13.6)	13.5 (11.5-15.4)	12.6 (10.6-14.5)	11.5 (9.5-13.5)	10.6 (8.6-12.6)	10.2 (8.2-12.1)	−2.1	−17.3	.29	
College degree or higher	6.4 (4.5-8.4)	5.9 (4.0-7.9)	5.5 (3.6-7.5)	5.5 (3.5-7.4)	6.3 (4.3-8.2)	6.0 (4.1-8.0)	6.3 (4.3-8.3)	5.7 (3.7-7.7)	−0.7	−11.6	.83	
Race and ethnicity–related discrimination												.18
Less than high school	2.7 (0.7-4.6)	3.6 (1.6-5.5)	4.7 (2.8-6.7)	7.0 (5.0-9.0)	5.6 (3.6-7.5)	4.1 (2.2-6.1)	2.2 (0.2-4.1)	3.6 (1.6-5.6)	0.9	34.7	.73	
High school or GED	2.9 (0.9-4.9)	3.8 (1.8-5.7)	4.0 (2.1-6.0)	4.3 (2.3-6.2)	5.3 (3.3-7.2)	3.2 (1.2-5.1)	4.1 (2.1-6.0)	3.8 (1.9-5.8)	0.9	32.2	.34	
Some college or technical school	4.6 (2.7-6.6)	4.1 (2.2-6.1)	4.5 (2.6-6.5)	4.7 (2.7-6.7)	5.5 (3.5-7.4)	5.4 (3.5-7.4)	4.9 (2.9-6.8)	4.8 (2.9-6.8)	0.2	4.2	.17	
College degree or higher	3.6 (1.6-5.6)	3.4 (1.5-5.4)	3.3 (1.4-5.3)	4.3 (2.3-6.3)	5.1 (3.2-7.1)	5.0 (3.0-6.9)	4.3 (2.4-6.3)	4.6 (2.7-6.6)	1.0	28.6	<.001	
Any ACE												.05
Less than high school	54.3 (52.4-56.3)	48.9 (46.9-50.8)	44.5 (42.6-46.5)	39.6 (37.7-41.6)	44.9 (42.9-46.8)	40.5 (38.5-42.4)	43.6 (41.7-45.6)	43.4 (41.4-45.3)	−11.0	−20.2	.007	
High school or GED	61.8 (59.8-63.8)	58.2 (56.2-60.1)	54.0 (52.0-56.0)	53.5 (51.6-55.5)	52.7 (50.7-54.7)	49.1 (47.2-51.1)	53.6 (51.6-55.6)	53.5 (51.5-55.4)	−8.3	−13.5	<.001	
Some college or technical school	58.7 (56.8-60.7)	56.4 (54.4-58.4)	50.6 (48.7-52.6)	52.7 (50.8-54.7)	50.0 (48.1-52.0)	49.5 (47.5-51.4)	51.0 (49.1-53.0)	48.3 (46.4-50.3)	−10.4	−17.7	<.001	
College degree or higher	32.8 (30.8-34.8)	31.8 (29.8-33.8)	29.1 (27.2-31.1)	29.3 (27.3-31.2)	29.3 (27.3-31.3)	28.3 (26.4-30.3)	28.7 (26.7-30.6)	27.6 (25.7-29.6)	−5.1	−15.7	<.001	
≥4 ACEs												.97
Less than high school	7.1 (5.1-9.0)	4.3 (2.4-6.3)	6.9 (4.9-8.8)	6.1 (4.1-8.0)	5.2 (3.3-7.2)	4.6 (2.7-6.6)	5.9 (3.9-7.8)	5.2 (3.2-7.1)	−1.9	−26.9	.31	
High school or GED	9.1 (7.2-11.1)	9.2 (7.2-11.2)	7.9 (6.0-9.9)	9.4 (7.4-11.3)	8.0 (6.1-10.0)	6.4 (4.5-8.4)	7.7 (5.8-9.7)	8.1 (6.2-10.1)	−1.0	−11.0	.04	
Some college or technical school	9.4 (7.5-11.4)	7.0 (5.1-9.0)	8.8 (6.8-10.7)	8.8 (6.8-10.7)	7.7 (5.8-9.7)	7.2 (5.2-9.1)	7.9 (5.9-9.8)	7.5 (5.5-9.4)	−2.0	−20.8	.05	
College degree or higher	3.1 (1.1-5.1)	3.0 (1.1-5.0)	2.9 (1.0-4.9)	2.4 (0.4-4.3)	2.6 (0.7-4.6)	2.8 (0.9-4.8)	2.6 (0.7-4.6)	2.7 (0.7-4.6)	−0.4	−13.9	.11	

Abbreviations: ACE, adverse childhood experience; GED, General Educational Development.

a All prevalences are survey-weighted. Data source: US Census Bureau.^13^

b Absolute difference represents the difference between the prevalence estimate in 2016 and 2023.

c Relative difference, expressed as the percentage change between 2016 and 2023, is calculated by dividing the absolute difference by the 2016 estimate and multiplying by 100.

d *P* for trend within group tests whether ACE prevalence changed linearly over time within each education category.

e *P* for trend between group tests whether linear time trends in ACE prevalence differed across education categories.

Financial hardship declined across all education groups (all *P*-trend < .001) but remained substantially higher among children living with parents with less than a high school education than among those with a college degree or higher in 2023. Parental imprisonment and parental divorce also showed overall downward trends, with prevalence remaining consistently higher among children with less-educated parents.

The prevalence of ≥4 ACEs showed a clear educational gradient, with modest declines in some groups, but persistent differences across all years. Domestic violence, neighborhood violence, and parental drug use showed minimal or inconsistent changes, while parental mental illness and race and ethnicity–related discrimination showed modest increases or remained stable over time.

### ACE Prevalence by Family Income (FPL)

Across all years, children in lower-income families consistently experienced a higher prevalence of most ACEs compared with those in higher-income groups (Table 5). Financial hardship declined across all income groups (all *P*-trend < .001), with larger reductions among children at <100% FPL than among those at ≥400% FPL. Despite these declines, prevalence remained substantially higher among lower-income children in 2023. Parental imprisonment and parental divorce also declined across most groups, with larger reductions among lower-income children but persistent disparities over time.

**Table 5.** Trends in Age-Standardized Prevalence of ACEs by Federal Poverty Level (FPL), United States, 2016-2023.^a^

ACE	2016	2017	2018	2019	2020	2021	2022	2023	Absolute difference^b^	Relative difference^c^	*P* for trend within FPL^d^	*P* for trend between FPL^e^
	% (95% CI)	% (95% CI)	% (95% CI)	% (95% CI)	% (95% CI)	% (95% CI)	% (95% CI)	% (95% CI)				
Financial hardship												.002
0%-99%	47.1 (45.1-49.0)	39.4 (37.4-41.3)	27.3 (25.4-29.3)	28.7 (26.8-30.7)	26.6 (24.7-28.6)	25.0 (23.0-26.9)	30.0 (28.0-31.9)	28.1 (26.2-30.1)	−18.9	−40.2	<.001	
100%-199%	35.8 (33.9-37.8)	34.2 (32.3-36.2)	24.8 (22.8-26.7)	24.2 (22.2-26.1)	20.6 (18.7-22.6)	19.6 (17.6-21.5)	23.1 (21.1-25.0)	24.0 (22.1-26.0)	−11.8	−33.0	<.001	
200%-399%	22.4 (20.5-24.4)	20.7 (18.8-22.7)	13.2 (11.2-15.1)	13.3 (11.3-15.2)	9.7 (7.7-11.6)	8.9 (7.0-10.9)	11.4 (9.5-13.4)	11.5 (9.6-13.5)	−10.9	−48.5	<.001	
≥400%	6.2 (4.2-8.1)	6.1 (4.2-8.1)	3.2 (1.2-5.1)	3.0 (1.0-5.0)	2.0 (0.1-4.0)	2.0 (0.0-3.9)	2.8 (0.8-4.7)	2.7 (0.8-4.7)	−3.4	−55.8	<.001	
Parental divorce												.01
0%-99%	37.2 (35.3-39.2)	34.3 (32.4-36.3)	32.7 (30.7-34.6)	35.5 (33.6-37.5)	32.7 (30.7-34.6)	29.9 (27.9-31.9)	31.4 (29.4-33.4)	30.4 (28.4-32.3)	−6.9	−18.5	<.001	
100%-199%	29.5 (27.6-31.5)	28.2 (26.2-30.1)	29.8 (27.8-31.8)	27.7 (25.8-29.7)	29.2 (27.2-31.1)	27.2 (25.2-29.1)	28.7 (26.7-30.6)	27.4 (25.4-29.4)	−2.1	−7.3	.23	
200%-399%	21.8 (19.9-23.8)	22.0 (20.1-24.0)	22.5 (20.5-24.4)	22.5 (20.5-24.4)	23.2 (21.2-25.1)	22.4 (20.5-24.4)	21.6 (19.7-23.6)	21.0 (19.0-22.9)	−0.9	−3.9	.43	
≥400%	14.7 (12.8-16.7)	13.1 (11.1-15.0)	13.5 (11.6-15.5)	13.1 (11.2-15.1)	13.0 (11.0-14.9)	12.8 (10.8-14.7)	12.1 (10.2-14.1)	11.7 (9.7-13.7)	−3.0	−20.6	<.001	
Death of parent												.09
0%-99%	5.2 (3.2-7.2)	5.7 (3.7-7.6)	4.8 (2.9-6.8)	4.1 (2.1-6.1)	4.9 (2.9-6.8)	3.6 (1.6-5.5)	4.3 (2.3-6.2)	4.8 (2.9-6.8)	−0.4	−7.0	.09	
100%-199%	3.2 (1.3-5.2)	3.9 (2.0-5.9)	3.9 (1.9-5.8)	3.8 (1.9-5.8)	3.5 (1.6-5.5)	4.3 (2.3-6.3)	3.3 (1.4-5.3)	4.3 (2.3-6.2)	1.0	32.5	.33	
200%-399%	3.3 (1.3-5.2)	3.1 (1.2-5.1)	2.8 (0.8-4.7)	2.9 (1.0-4.9)	2.5 (0.6-4.5)	2.6 (0.6-4.5)	3.0 (1.0-4.9)	2.7 (0.8-4.7)	−0.6	−17.2	.23	
≥400%	1.8 (−0.1 to 3.8)	2.1 (0.1-4.0)	1.8 (−0.1 to 3.8)	1.7 (−0.2 to 3.7)	1.3 (−0.7 to 3.3)	1.9 (−0.1 to 3.8)	1.6 (−0.4 to 3.5)	1.3 (−0.7 to 3.2)	−0.6	−31.2	.01	
Parental imprisonment												.49
0%-99%	15.3 (13.4-17.3)	12.9 (10.9-14.8)	15.8 (13.8-17.7)	13.6 (11.6-15.5)	13.8 (11.8-15.7)	11.7 (9.7-13.6)	12.9 (10.9-14.8)	9.9 (7.9-11.8)	−5.4	−35.4	<.001	
100%-199%	10.5 (8.5-12.4)	9.9 (8.0-11.9)	10.0 (8.0-12.0)	11.5 (9.5-13.4)	9.0 (7.1-11.0)	8.3 (6.3-10.2)	8.2 (6.3-10.2)	9.2 (7.2-11.2)	−1.3	−12.1	.007	
200%-399%	6.4 (4.5-8.4)	6.2 (4.2-8.1)	5.0 (3.0-7.0)	5.7 (3.7-7.6)	5.7 (3.8-7.7)	5.7 (3.8-7.7)	5.2 (3.2-7.1)	5.2 (3.2-7.1)	−1.3	−19.9	.06	
≥400%	2.5 (0.5-4.4)	2.2 (0.3-4.2)	2.5 (0.6-4.5)	2.1 (0.2-4.1)	1.7 (−0.3 to 3.7)	2.2 (0.2-4.2)	1.9 (−0.0 to 3.9)	1.8 (−0.1 to 3.8)	−0.6	−24.9	.014	
Domestic violence												.36
0%-99%	10.1 (8.2-12.1)	6.4 (4.4-8.3)	10.7 (8.7-12.7)	10.3 (8.3-12.2)	9.3 (7.4-11.3)	7.3 (5.3-9.2)	9.7 (7.7-11.6)	7.1 (5.2-9.1)	−3.0	−29.7	.12	
100%-199%	6.9 (5.0-8.9)	7.0 (5.0-8.9)	7.3 (5.4-9.3)	6.9 (4.9-8.9)	5.9 (4.0-7.9)	5.7 (3.8-7.7)	7.2 (5.3-9.2)	7.3 (5.4-9.3)	0.4	6.0	.78	
200%-399%	4.5 (2.6-6.5)	4.9 (3.0-6.9)	4.7 (2.7-6.6)	4.8 (2.9-6.8)	5.0 (3.0-6.9)	5.1 (3.1-7.0)	4.8 (2.9-6.8)	5.0 (3.0-6.9)	0.4	9.1	.48	
≥400%	2.4 (0.5-4.4)	2.4 (0.5-4.4)	2.5 (0.5-4.4)	2.6 (0.6-4.5)	2.3 (0.3-4.2)	2.2 (0.3-4.2)	2.3 (0.3-4.2)	2.2 (0.2-4.1)	−0.2	−9.6	.21	
Neighborhood violence												.67
0%-99%	7.0 (5.0-8.9)	6.2 (4.2-8.2)	8.1 (6.2-10.1)	6.8 (4.8-8.7)	7.3 (5.4-9.3)	6.3 (4.4-8.3)	7.4 (5.4-9.4)	5.5 (3.6-7.5)	−1.4	−20.5	.37	
100%-199%	4.5 (2.6-6.5)	5.1 (3.1-7.1)	4.9 (2.9-6.9)	6.4 (4.5-8.4)	5.1 (3.2-7.1)	4.9 (3.0-6.9)	5.0 (3.0-6.9)	5.1 (3.1-7.0)	0.5	11.7	.75	
200%-399%	3.1 (1.1-5.1)	3.0 (1.1-5.0)	3.0 (1.0-5.0)	3.2 (1.2-5.1)	3.1 (1.2-5.1)	2.6 (0.6-4.5)	3.1 (1.1-5.0)	3.6 (1.7-5.6)	0.5	16.8	.51	
≥400%	1.6 (−0.4 to 3.5)	2.0 (−0.0 to 3.9)	1.7 (−0.3 to 3.6)	1.6 (−0.3 to 3.6)	1.8 (−0.2 to 3.7)	1.8 (−0.2 to 3.7)	1.6 (−0.4 to 3.5)	1.7 (−0.3 to 3.6)	0.1	5.0	.67	
Parental mental illness												.36
0%-99%	10.5 (8.5-12.4)	7.9 (6.0-9.9)	8.9 (6.9-10.8)	11.0 (9.1-13.0)	9.5 (7.5-11.4)	8.7 (6.8-10.7)	11.3 (9.4-13.3)	8.6 (6.7-10.6)	−1.9	−17.7	.81	
100%-199%	8.7 (6.7-10.7)	8.5 (6.6-10.5)	8.7 (6.7-10.6)	10.7 (8.7-12.6)	10.2 (8.2-12.1)	8.2 (6.2-10.2)	11.1 (9.1-13.0)	10.1 (8.2-12.1)	1.4	16.3	.02	
200%-399%	7.6 (5.7-9.6)	7.9 (5.9-9.9)	8.1 (6.2-10.1)	8.7 (6.7-10.6)	8.2 (6.2-10.1)	8.9 (6.9-10.8)	9.4 (7.4-11.3)	9.4 (7.4-11.3)	1.7	22.4	.002	
≥400%	5.5 (3.5-7.4)	4.6 (2.6-6.6)	5.6 (3.7-7.6)	6.2 (4.2-8.1)	6.2 (4.2-8.1)	6.0 (4.1-8.0)	5.8 (3.8-7.7)	6.2 (4.2-8.2)	0.7	13.5	.006	
Parental drug use												.31
0%-99%	12.3 (10.3-14.2)	10.0 (8.0-11.9)	9.7 (7.7-11.6)	11.4 (9.4-13.4)	9.9 (7.9-11.9)	9.1 (7.1-11.0)	10.8 (8.8-12.7)	9.2 (7.3-11.2)	−3.1	−24.9	.07	
100%-199%	10.7 (8.7-12.6)	9.1 (7.1-11.0)	9.9 (7.9-11.8)	11.9 (9.9-13.9)	10.3 (8.3-12.2)	8.6 (6.7-10.6)	10.6 (8.6-12.5)	10.7 (8.7-12.6)	0.0	0.2	.77	
200%-399%	8.7 (6.7-10.7)	8.4 (6.4-10.3)	8.3 (6.3-10.3)	9.2 (7.2-11.1)	9.5 (7.5-11.4)	9.2 (7.3-11.2)	8.4 (6.4-10.4)	8.4 (6.4-10.4)	−0.3	−3.5	.86	
≥400%	5.8 (3.9-7.8)	5.0 (3.1-7.0)	5.3 (3.4-7.3)	4.6 (2.6-6.5)	5.3 (3.3-7.2)	5.2 (3.3-7.2)	5.3 (3.3-7.2)	4.9 (2.9-6.8)	−1.0	−17.0	.24	
Race and ethnicity–related discrimination												.004
0%-99%	4.8 (2.8-6.8)	5.0 (3.0-6.9)	6.0 (4.1-8.0)	5.9 (3.9-7.9)	6.9 (5.0-8.9)	5.1 (3.1-7.0)	4.2 (2.3-6.2)	4.9 (2.9-6.8)	0.1	1.5	.56	
100%-199%	4.4 (2.5-6.4)	4.0 (2.0-6.0)	3.9 (1.9-5.9)	5.7 (3.8-7.7)	6.3 (4.4-8.3)	4.6 (2.6-6.5)	4.8 (2.8-6.7)	4.7 (2.7-6.6)	0.3	5.9	.21	
200%-399%	3.5 (1.5-5.4)	3.7 (1.7-5.7)	3.2 (1.3-5.2)	5.2 (3.3-7.2)	5.0 (3.0-6.9)	5.2 (3.2-7.2)	4.6 (2.6-6.6)	4.8 (2.9-6.8)	1.3	38.4	<.001	
≥400%	2.3 (0.4-4.3)	2.6 (0.6-4.6)	3.0 (1.1-5.0)	2.8 (0.8-4.7)	3.8 (1.9-5.8)	3.8 (1.9-5.8)	3.5 (1.5-5.4)	3.7 (1.7-5.7)	1.4	59.1	<.001	
Any ACE												.004
0%-99%	67.3 (65.3-69.3)	62.0 (60.1-64.0)	54.5 (52.5-56.5)	57.4 (55.4-59.4)	56.4 (54.4-58.4)	52.6 (50.6-54.6)	57.3 (55.3-59.2)	54.6 (52.7-56.6)	−12.7	−18.8	<.001	
100%-199%	57.7 (55.8-59.7)	54.4 (52.5-56.4)	51.2 (49.2-53.1)	49.2 (47.2-51.2)	49.8 (47.9-51.8)	46.2 (44.2-48.2)	50.7 (48.7-52.6)	48.6 (46.7-50.6)	−9.1	−15.8	<.001	
200%-399%	43.7 (41.8-45.7)	43.8 (41.9-45.8)	38.8 (36.8-40.8)	39.9 (37.9-41.8)	39.1 (37.2-41.1)	37.6 (35.7-39.6)	38.8 (36.9-40.8)	37.6 (35.6-39.5)	−6.2	−14.1	<.001	
≥400%	25.9 (23.9-27.9)	24.4 (22.5-26.4)	24.2 (22.2-26.1)	23.6 (21.6-25.5)	23.2 (21.2-25.1)	23.4 (21.5-25.4)	22.4 (20.4-24.4)	22.3 (20.4-24.3)	−3.6	−13.8	<.001	
≥4 ACEs												.35
0%-99%	12.1 (10.1-14.0)	8.3 (6.4-10.3)	10.8 (8.9-12.8)	10.4 (8.5-12.4)	9.5 (7.5-11.5)	8.3 (6.3-10.3)	9.5 (7.5-11.4)	8.2 (6.2-10.1)	−3.9	−32.3	.003	
100%-199%	7.9 (5.9-9.8)	8.5 (6.5-10.4)	8.0 (6.0-9.9)	8.7 (6.8-10.7)	7.2 (5.3-9.2)	5.8 (3.8-7.8)	7.4 (5.4-9.3)	7.7 (5.8-9.7)	−0.1	−1.6	.09	
200%-399%	4.8 (2.9-6.8)	4.5 (2.6-6.5)	4.3 (2.3-6.3)	4.4 (2.5-6.4)	4.6 (2.6-6.6)	4.5 (2.6-6.5)	4.2 (2.2-6.1)	4.6 (2.7-6.6)	−0.2	−4.2	.70	
≥400%	2.0 (0.0-4.0)	1.9 (−0.1 to 3.8)	1.9 (−0.1 to 3.8)	1.4 (−0.5 to 3.4)	1.3 (−0.6 to 3.3)	1.8 (−0.1 to 3.8)	1.8 (−0.2 to 3.8)	1.6 (−0.4 to 3.5)	−0.4	−20.6	.20	

Abbreviation: ACE, adverse childhood experience.

a All prevalences are survey-weighted. Data source: US Census Bureau.^13^

b Absolute difference represents the difference between the prevalence estimate in 2016 and 2023.

c Relative difference, expressed as the percentage change between 2016 and 2023, is calculated by dividing the absolute difference by the 2016 estimate and multiplying by 100.

d *P* for trend within group tests whether ACE prevalence changed linearly over time within each FPL category.

e *P* for trend between group tests whether linear time trends in ACE prevalence differed across FPL categories.

The prevalence of ≥4 ACEs showed a clear income gradient, declining most notably among children at <100% FPL, from 12.1% in 2016 to 8.2% in 2023. In comparison, prevalence remained relatively stable among children in higher-income groups, including those at 100% to 199% FPL (from 7.9% to 7.7%) and 200% to 399% FPL (from 4.8% to 4.6%), and declined modestly among those at ≥400% FPL (from 2.0% to 1.6%). Domestic violence, neighborhood violence, and parental drug use showed minimal or inconsistent changes, whereas parental mental illness and race and ethnicity–related discrimination showed modest increases or remained stable.

## Discussion

Our analysis provides national-level temporal trends and social inequalities in age-standardized ACE prevalences in the United States using the most up-to-date NSCH data. Using age-standardized prevalence estimates from repeated cross-sectional NSCH data, this study provides comparable estimates of ACE prevalence across survey years and population subgroups. Overall, a central pattern in the analysis was a substantial decline in material and household instability-related ACEs, including financial hardship, parental imprisonment, parental divorce, and ≥4 ACEs. In contrast, we observed stability or modest increases in parental mental illness and race and ethnicity–related discrimination. Despite downward trends in some ACEs, social inequities in ACE exposure remained pronounced among Black children, children in native-born families, those living with caregiver(s) other than parents, and those living in families with lower income or education levels.

The findings reveal a clear divergence in temporal trends across ACE types. We found significant downward trends, particularly in financial hardship, parental imprisonment, parental divorce, and the severity of ACE. These ACEs reflect material conditions and household dysfunctions, and their declines may partly reflect improvements in macroeconomic conditions, expansions in social safety-net programs (eg, Supplemental Nutrition Assistance Program, Earned Income Tax Credit, and Child Tax Credit during the COVID-19 pandemic), and declines in incarceration rates in the United States.^18-21^ We note that these interpretations remain speculative, given the absence of relevant policy measures in the NSCH.

Furthermore, the observed decline in parental drug use should be interpreted cautiously. Changes in social norms and the increasing legalization of cannabis in the United States may have influenced how parents/caregivers perceive and report “drug use,” potentially contributing to the observed trend independent of actual changes in substance use. Because this measure is based on parent-reported information, it may be particularly sensitive to shifts in reporting behavior over time.^22^

Apart from macroeconomic policy responses, other socio-cultural and structural factors may also have contributed to the overall downward trends in ACEs. Shifting parenting practices—characterized by greater use of reasoning and emotional responsiveness and less reliance on harsh discipline, especially among recent cohorts of parents—have been reported to positively shape children’s psychosocial environment and lower the risk of ACEs.^23,24^ Furthermore, recent expansion of state-led ACE screening and prevention strategies, supported by financial assistance, pediatric provider incentives, and growing awareness of positive parenting, may have contributed to reductions in ACE exposure at the population level.^25-27^ On the other hand, psychosocial and structural adversities, including parental mental illness and race and ethnicity–related discrimination, showed modest increases or little change over time. The observed increase in parental mental illness should be interpreted with caution, as it may partly reflect increased awareness of mental health conditions, greater access to screening and diagnosis, and changing reporting behaviors over time, rather than a true increase in underlying prevalence. These patterns suggest that such exposures are deeply rooted in social and structural contexts and are less sensitive to short-term economic or policy shifts.^3,5^

Our analysis found persistent racial and ethnic and nativity-based differences in ACE exposure across years. Black children consistently experienced the highest prevalence of several ACEs, particularly financial hardship, parental imprisonment, neighborhood violence, and race and ethnicity–related discrimination. Despite the largest absolute reductions in several ACEs among Black children—particularly financial hardship and parental imprisonment—prevalence remained substantially higher than among White children, and for some outcomes, Hispanic children. The Black–White gap in parental imprisonment, neighborhood violence, and race and ethnicity–related discrimination was particularly pronounced and persistent. Although some absolute reductions were larger among Black children, formal interaction tests suggested that differences in temporal trends were outcome-specific rather than uniform across ACE domains.

Furthermore, children living with caregivers other than parents faced disproportionately high exposure to multiple ACEs, including financial hardship and parental imprisonment. These patterns are consistent with prior NSCH-based studies that documented entrenched social patterning in childhood adversity. Nativity-based patterns—showing lower ACE burden among children of foreign-born parents—align with the immigrant paradox, which largely posits that, despite having socioeconomic disadvantage, immigrant families exhibit protective cultural, familial, or social buffering. However, differences in temporal trends by parental nativity were limited to selected ACEs, particularly financial hardship and death of parent, whereas most other ACEs showed no statistical evidence of differing trends across nativity groups.

We observed persistent socioeconomic inequalities in ACE exposure over time, with children from low-income and less-educated families consistently reporting a higher burden of most ACEs. These inequalities were most pronounced for financial hardship, parental imprisonment, and severe ACE exposure (≥4 ACEs), where prevalence remained substantially higher among socioeconomically disadvantaged groups. Although several ACEs declined within income and education groups, there was little evidence that trends differed between groups, indicating that overall reductions did not meaningfully narrow socioeconomic disparities. These results align with previous studies showing persistent income and education gradients in ACE exposure, particularly for ACE severity and material hardship–related ACEs. Most prior work has been cross-sectional, limiting insight into temporal patterns.^18,28,29^ For example, children experiencing ≥4 ACEs are disproportionately concentrated in lower-income families, and county-level poverty has been linked to increasing child maltreatment over time.^30^ By leveraging repeated cross-sectional data over 8 years, our study extends this literature by showing that, despite overall declines in some ACEs, socioeconomic inequalities remain largely unchanged.

### Limitations

Some limitations should be considered when interpreting our results. First, ACE measurement is subject to response bias. Parents/caregivers might underreport certain ACEs that directly implicate themselves, including domestic violence, parental mental illness, and parental substance use.^31,32^ Second, the NSCH ACE measures do not capture timing or chronicity, nor do they distinguish between recent and earlier exposure, which may limit the ability to explore life-course dynamics. Third, some ACEs (eg, race and ethnicity–related unfair treatment/discrimination and neighborhood violence) reflect the parent/caregiver’s perception rather than objective exposure. These limitations may lead to underestimation or overestimation of the prevalence estimates. Fourth, our analyses were limited to the race and ethnicity categories available in the harmonized Data Resource Center files and, therefore, could not assess heterogeneity within broader racial and ethnic groups. Future research using more granular racial and ethnic classifications may help identify important disparities in ACE exposure that are not captured by aggregated categories. Fifth, because individual ACEs are often correlated, separate analyses of each ACE involved multiple statistical comparisons that may have increased the likelihood of chance findings. Accordingly, individual *P* values should be interpreted cautiously, and greater emphasis should be placed on the overall consistency, direction, and magnitude of observed trends across years and population subgroups. Finally, the ACE measures available in the NSCH capture whether a child has ever experienced each adversity by the time of the survey and do not provide information on the timing, duration, or frequency of exposure. Although we age-standardized prevalence estimates to improve comparability across years and sociodemographic groups, age standardization cannot fully account for differences in exposure duration across childhood. As a result, some variation in ACE prevalence may reflect differences in how long children have been at risk of adversity.

Despite these limitations, our analysis has several important strengths. This study is among the first national ACE trend analyses to use age standardization to estimate ACE prevalence. Failure to account for differences in age composition across survey years may reduce comparability of prevalence estimates because older children have had a greater opportunity to experience lifetime ACEs. The direct age standardization we applied improves comparability across survey years and population subgroups, addressing a key limitation of prior ACE trend studies. Unlike many previous national trend studies that primarily emphasized cumulative ACE measures, we examined trends across 9 individual ACE items in addition to commonly used summary indicators. We also examined multiple social inequalities, including race and ethnicity, parental nativity, income, and education.

## Conclusions

Significant reductions in several ACEs were observed between 2016 and 2023. Material and household instability–related ACEs declined most, while psychosocial and structural adversities showed little improvement. Although racial and ethnic– and nativity-related disparities narrowed for some ACEs, income- and education-related differences persisted across most ACEs. These findings underscore the need for structural and equity-focused approaches to ACE prevention beyond overall reductions in prevalence.
